# Metabolomics-Driven Insights into Biomarkers for Poor Ovarian Response: A Narrative Review

**DOI:** 10.3390/biomedicines13010214

**Published:** 2025-01-16

**Authors:** Anastasios Potiris, Sofoklis Stavros, Eleni Alyfanti, Nikolaos Machairiotis, Eirini Drakaki, Athanasios Zikopoulos, Efthalia Moustakli, Charikleia Skentou, Peter Drakakis, Ekaterini Domali

**Affiliations:** 1 Third Department of Obstetrics and Gynecology, University General Hospital “ATTIKON”, Medical School, National and Kapodistrian University of Athens, 124 62 Athens, Greece; sfstavrou@med.uoa.gr (S.S.); nikolaosmachairiotis@gmail.com (N.M.); thanzik92@gmail.com (A.Z.); pdrakakis@med.uoa.gr (P.D.); 2 Medical School, National and Kapodistrian University of Athens, 115 27 Athens, Greece; elenalyfanti@med.uoa.gr; 3 First Department of Obstetrics and Gynecology, Alexandra Hospital, Medical School, National and Kapodistrian University of Athens, 115 28 Athens, Greece; eirinidrak@med.uoa.gr (E.D.); kdomali@yahoo.fr (E.D.); 4 Laboratory of Medical Genetics, Medical School, University of Ioannina, 451 10 Ioannina, Greece; thaleia.moustakli@gmail.com; 5 Department of Obstetrics and Gynecology, Medical School, University of Ioannina, 451 10 Ioannina, Greece; haraskentou@uoi.gr

**Keywords:** poor ovarian response (POR), diminished ovarian reserve (DOR), metabolomics, follicular fluid metabolomic profiles, biomarkers

## Abstract

Poor ovarian response (POR) remains a significant challenge in the field of assisted reproductive technology (ART), as the quantity and quality of oocytes retrieved directly influence embryo implantation, clinical pregnancy, and live birth rates. Metabolomics has become a valuable tool for elucidating the molecular mechanisms underlying diminished ovarian reserve (DOR) and POR. This review aims to synthesize findings from metabolomic studies examining metabolite expression patterns in serum and follicular fluid samples from women with POR. A literature search was performed using the Medline/PubMed and Scopus databases, employing keywords related to metabolomics and POR. In total, nine studies met the inclusion criteria for this review. These studies identified several metabolites with differential expression in serum and follicular fluid samples between women with normal ovarian response and those with POR. Although the metabolomic profiles varied significantly among studies, consistent alterations in prostaglandin related metabolites were observed in two of the nine studies reviewed. These findings suggest that, pending further validation, these metabolites may serve as potential biomarkers for ovarian response. Metabolomics has significantly advanced our understanding of the mechanisms underlying ovarian function and holds promise for identifying effective biomarkers that could improve the prediction and management of POR.

## 1. Introduction

Poor ovarian response (POR) refers to an insufficient or suboptimal response to controlled ovarian stimulation (COS) during assisted reproductive technology (ART) cycles, resulting in a low yield of oocytes and presenting significant challenges for in vitro fertilization (IVF) success. POR is widely regarded as an indicator of poor or reduced ovarian reserve and affects approximately 9–24% of patients undergoing ovarian stimulation for IVF [1]. The first standardized criteria for defining and predicting POR were established with the Bologna criteria, developed by the European Society of Human Reproduction and Embryology (ESHRE) [2]. In 2016, the POSEIDON classification was introduced as a more refined approach, incorporating factors such as patient age, ovarian reserve, and the anticipated quantity and quality of oocytes [3]. This classification reduces population heterogeneity, offering a more individualized framework for managing and counseling POR patients [4].

Diminished ovarian reserve (DOR), also known as poor ovarian reserve (POR), describes a condition in which women of reproductive age have a reduced quantity or quality of oocytes and is identified through biomarkers such as anti-Müllerian hormone (AMH) levels and antral follicle count (AFC), which are predictive of suboptimal response to ovarian stimulation [5]. Unfortunately, DOR is linked to several challenges during in vitro fertilization (IVF) cycles, including reduced ovarian response, increased cycle cancelation rates, fewer oocytes and embryos, lower embryo implantation and clinical pregnancy rates, lower live birth rates, and higher rates of miscarriage and embryo aneuploidy [6,7,8,9]. Reduced ovarian function can occur from a variety of factors, including age-related depletion of ovarian follicles, genetic predispositions, endometriosis, autoimmune disorders, metabolic and enzymatic diseases, cancer treatments, lifestyle, or, in some cases, idiopathic origins [10,11,12]. The multifactorial etiology of POR adds considerable complexity, as the underlying pathogenic mechanisms remain only partially understood [10,13]. This lack of clarity hinders the identification of reliable predictive biomarkers and the development of targeted, effective IVF strategies for individuals affected by POR.

Metabolomics—the comprehensive analysis of metabolites in biofluids, cells, and tissues—has become a powerful tool for biomarker discovery and for elucidating the biochemical pathways and mechanisms underlying physiological and pathological processes [14]. Advances in data analysis, cutting-edge analytical technologies, and the integration of complementary biological methods now enable metabolomic studies to reveal the broader, systems-level impacts of metabolites [14,15]. The methodologies for recovering and identifying metabolites include untargeted and targeted mass spectrometry-based approaches. Untargeted metabolomics seeks to capture the widest possible range of metabolites within a sample, without prior knowledge of the metabolome. In contrast, targeted metabolomics offers higher sensitivity and selectivity by focusing on specific metabolites and pathways, leveraging pre-existing knowledge to develop and optimize methods for these particular analytes [16,17].

In the field of ART, metabolomics has become an increasingly valuable tool for providing insights into reproductive biology and improving clinical outcomes. Key applications include assessing oocyte and embryo quality through the analysis of biofluids like follicular fluid and spent culture media, as well as offering a non-invasive approach to embryo selection by profiling metabolites in culture media [18,19,20,21]. Additionally, metabolomics contributes to predicting ovarian response to controlled ovarian stimulation and aids in understanding metabolic disruptions associated with several reproductive pathologies, such as polycystic ovary syndrome (PCOS) and endometriosis [20,22,23].

This review aims to summarize findings from metabolomic studies investigating metabolite expression patterns in serum or follicular fluid samples of women with poor ovarian response (POR), with the goals of enhancing understanding of the mechanisms underlying POR and identifying biomarkers with the potential to improve ART strategies and outcomes in this patient group.

## 2. Materials and Methods

To conduct this narrative review, a search of the available literature was performed in the Medline/PubMed and Scopus up to October 2024. Keywords relevant to metabolomics, such as “metabolomic analysis”, “metabolome”, and “metabolomic profiles”, were used alongside with terms associated with poor ovarian response (POR), including “poor ovarian responders”, “poor ovarian reserve”, “decreased ovarian reserve”, and “biomarkers of ovarian response”. These terms were applied independently or in combination with Boolean operators (OR, AND) to refine the search. Only studies published after 2013 with English titles and abstracts were initially considered. Exclusions were applied to studies that lacked detailed sample information, or examined samples based on cell lines or animal models.

During the screening process, the titles and abstracts of 18 studies were screened independently by two reviewers (A.P. and E.A.). A subsequent full-text assessment was conducted. If a study was selected by only one reviewer, a third reviewer (S.S.) made the final decision. Ultimately, 13 studies proceeded to full-text assessment, and after detailed evaluation, 9 articles were included in this review. Data extraction was performed by one reviewer (E.A.) and verified by a second reviewer (A.P.). A formal risk of bias and quality assessment was not performed due to the narrative nature of this review.

## 3. Results

### 3.1. Comparison of Metabolomic Profiles in Serum Samples Between Women with Poor Ovarian Reserve and Women with Normal Ovarian Reserve

The pilot study by Borges Jr. et al. was the first to demonstrate that serum metabolites could serve as predictive molecular markers for assessing ovarian response to controlled ovarian stimulation (COS). Using a hybrid liquid chromatography–mass spectrometry (LC-MS) technique with a quadrupole time-of-flight mass spectrometer (QTOF-MS) equipped with an Apollo II electrospray ion source (Bruker, Billerica, MA, USA) and coupled to a prominence ultra-fast liquid chromatography (UFLC) binary system (Shimadzu, Kyoto, Japan), the researchers analyzed the metabolomic profiles of serum samples from women with poor (PR), normal (NR), and high (HR) responses to ovarian stimulation treatment. Principal component analysis (PCA) was then applied to compare data from these three groups, identifying intrinsic clusters based on metabolic profiles. PCA effectively distinguished between PR, NR, and HR groups, and a total of 10 ions, more specific to the PR group than the NR group, were selected as potential COS response biomarkers. These ions are associated with fatty alcohols, amino acids, peptides and their analogs, quinones and hydroquinone lipids, steroidal glycosides, tertiary amines, triacylglycerols, and methoxyphenols [24].

In 2021, Song et al. employed an LC-MS-based untargeted metabolomics approach to characterize serum metabolic alterations between patients with poor ovarian reserve (POR) and women with normal ovarian reserve (NOR), aiming to identify potential biomarkers predictive of POR. The study identified 538 metabolites with significant differences between the two groups. Specifically, 1-naphthylamine, lidocaine, D-erythrulose 1-phosphate, diphenylamine, 2-arachidonoylglycerol, pyracarbolid, lanosterin, pelargonic acid, sebacic acid, and 2,4-dinitrophenol were significantly upregulated in POR patients (*p* < 0.001), while prostaglandin H2, cortexolone, tetracosanoic acid, and 5-hydroxymethyl-2-furancarboxaldehyde were downregulated (*p* < 0.01). Using advanced statistical and machine learning techniques, the researchers identified nine key metabolites—tetracosanoic acid, 2-arachidonoylglycerol, lidocaine, cortexolone, prostaglandin H2, 1-naphthylamine, 5-hydroxymethyl-2-furancarboxaldehyde, D-erythrulose 1-phosphate, and 2,4-dinitrophenol (*p* < 0.05)—as the most critical biomarkers distinguishing between POR and NOR groups. Furthermore, by integrating metabolomic profiling with support vector machine (SVM) and pathway analysis, the team found that the nicotinate and nicotinamide metabolism pathway—including metabolites such as L-aspartic acid, 6-hydroxynicotinate, maleic acid, and succinic acid semialdehyde—showed significant alterations in the POR group. This finding suggests a potential link between this pathway and ovarian reserve, offering new perspectives for improving the prediction and treatment of POR [25].

The research team of An et al., in 2024, utilized a large-scale LC-MS-based untargeted metabolomics approach to compare serum metabolite profiles between women with decreased ovarian reserve (DOR) and those with normal ovarian reserve (NOR) undergoing IVF, aiming to explore the effects of DOR on IVF outcomes and identify associated metabolic pathways. Results indicated that DOR was significantly linked to poorer IVF outcomes, as evidenced by a lower number of retrieved oocytes (*p* < 0.001), diminished embryo quality (*p* < 0.001), and reduced pregnancy and live birth rates (*p* < 0.001). Metabolomic analysis revealed notable differences between the two groups, with 82 metabolites showing significant changes, including various lipids, lipid molecules, organic acids, and their derivatives. Using binary logistic regression analysis, the team identified a panel of five metabolites—stearic acid, palmitic acid, PC(18:0/9:0(CHO)), PC(16:0/9:0(CHO)), and LysoPC(9:0(CHO)/0:0)—that distinguished effectively between the DOR and NOR groups and correlated strongly with IVF outcomes. Moreover, pathway analysis indicated that metabolic dysregulation in DOR was primarily linked to processes such as unsaturated fatty acid biosynthesis, linoleic acid metabolism, sphingolipid metabolism, aminoacyl-tRNA biosynthesis, alpha-linolenic acid metabolism, arginine biosynthesis, and the biosynthesis of phenylalanine, tyrosine, and tryptophan, as well as glycerophospholipid and phenylalanine metabolism. Notably, dysregulation of unsaturated fatty acid biosynthesis was particularly prominent, suggesting a potential target for therapeutic intervention. Finally, enrichment analysis showed that several DOR-associated metabolites are implicated in the pathogenesis of various human disorders, including argininosuccinic aciduria, short bowel syndrome, and ornithine transcarbamylase deficiency [26]. Table 1 summarizes the identified metabolites with differential expression in the serum of women with diminished ovarian reserve.

### 3.2. Comparison of Metabolomic Profiles in Follicular Fluid Samples Between Women with Poor Ovarian Reserve and Women with Normal Ovarian Reserve

In 2013, Cataldi et al. conducted an observational case–control study using matrix-assisted laser desorption/ionization mass spectrometry (MALDI-MS) to compare the lipid profiles in the follicular fluid of young women with poor ovarian response (POR) to those of normal responders (NR) undergoing ovarian stimulation for IVF. The study identified a total of 10 overexpressed ions. Among these, three ions from the phosphatidylcholine (PC) subclass were found at higher concentrations in the NR group. The remaining seven ions, found in higher concentrations in the POR group, potentially serve as lipid biomarkers for poor responders and belong to four main lipid subclasses: phosphatidylethanolamines (PE), phosphatidylglycerols (PG), phosphatidylinositols (PI), and diacylglycerols (DAG). These lipids play roles in several biological and molecular processes, including hormonal responses, oocyte production and quality, cell proliferation, and apoptosis. According to Cataldi et al., changes in the levels of these lipids could guide therapeutic interventions for women with poor ovarian response [27].

The team of de la Barca JMC et al., in 2017, conducted an observational study to assess differences in the follicular fluid (FF) metabolomic profiles of IVF patients with diminished (DOR) versus normal ovarian reserve (NOR). Using a targeted quantitative metabolomic approach, the team employed high-performance liquid chromatography coupled with tandem mass spectrometry and the Biocrates Absolute IDQ p180 kit, enabling the precise quantification of 136 metabolites, from which 23 sums and ratios were calculated. Samples were randomly split into training and validation sets. Multivariate models, including orthogonal partial least squares discriminant analysis (OPLS-DA) and least absolute shrinkage and selection operator (LASSO-LR), were developed and validated, identifying three key predictors: the total dimethylarginine-to-arginine ratio (Total DMA/Arginine), polyunsaturated choline plasmalogens (PUFA ae), and patient age. The FF profile in DOR patients showed significantly lower levels of polyunsaturated choline plasmalogens and methyl arginine transferase activity. Additionally, anti-Müllerian hormone (AMH) levels, antral follicle count (AFC), and the number of oocytes retrieved were positively associated with these three identified variables. Overall, the study underscores the impact of ovarian aging on FF composition, highlighting a distinct profile of reduced polyunsaturated choline plasmalogens and methyl transferase activity in DOR patients [28].

In 2021, Liang et al. utilized ultra-high performance liquid chromatography–tandem mass spectrometry (UHPLC-MS/MS) technology to detect oxylipin metabolites in the follicular fluid (FF) of patients with diminished ovarian reserve (DOR). Oxylipin metabolic profiles in the FF of DOR patients were compared with those of women with a normal ovarian reserve (NOR) undergoing in vitro fertilization (IVF). Following UHPLC-MS/MS analysis, principal component analysis (PCA) and orthogonal projections to latent structure discriminant analysis (OPLS-DA) were used to evaluate the metabolomic data. Results indicated that fifteen oxylipin metabolites, including ±20-HDoHE, ±5-iso PGF2α-VI, 12S-HHTrE, 15-deoxy-Δ12,14-PGJ2, 1a,1b-dihomo PGE2, 1a,1b-dihomo PGF2α, 20-COOH-AA, 20-HETE, 8S,15S-DiHETE, PGA2, PGD2, PGE1, PGF1α, PGF2α, and PGJ2, were significantly lower in the FF of DOR patients compared to the NOR group. Pathway enrichment analysis revealed that these fifteen downregulated metabolites were closely linked to the arachidonic acid (AA) metabolic pathway. Spearman’s correlation analysis further demonstrated that eight oxidized lipid metabolites were negatively correlated with FSH levels and positively correlated with antral follicle count (AFC). Additionally, AMH levels, the number of oocytes retrieved, MII oocytes, and fertilization rates showed positive correlations with nine specific metabolites, while only one metabolite was positively correlated with the number of high-quality embryos. Overall, the study suggests that differential oxylipin metabolites and the AA metabolic pathway play an important role in DOR-associated infertility. These findings may support the identification of biomarkers and enhance understanding of the oocyte microenvironment, offering potential pathways to improve oocyte quality in DOR patients [29].

The study conducted by Shen et al., in 2022, aimed to examine the differential expression of follicular fluid metabolites in diminished ovarian reserve (DOR), polycystic ovarian syndrome (PCOS), and normal ovarian response (NOR) groups. Metabolites identified via LC-MS/MS analysis were annotated using the KEGG database to explore metabolic pathway alterations associated with PCOS and DOR. A regression model combined with ROC analysis was employed to pinpoint potential biomarkers. The results revealed that, in the DOR group, five metabolites—(S)-nerolidol 3-O, S-japonin, 2-hydroxyestrone sulfate, pregnanediol-3-glucuronide, and 3-O-acetylepisamarcandin—were significantly reduced, while another five metabolites—isopropyl linoleate, DG (18:0/18:2(9Z,12Z)/0:0), mactraxanthin, DG (18:0/16:1(9Z)/0:0), and PE (16:1(9Z)/P-18:1(11Z))—were significantly increased compared to the NOR group. In the PCOS group, metabolites such as deoxyadenosine, (E)-1-O-cinnamoyl-beta-D-glucose, 6-(2-hydroxyethoxy)-6-oxohexanoic acid, aspartyl-lysine, and epidermin were downregulated, while 3,4-dehydrothiomorpholine-3-carboxylate, N-acetyl-S-(N-methylcarbamoyl) cysteine, L-NIL, umbelliferone, and soyasaponin aa were upregulated compared to the NOR group. KEGG pathway analysis showed that the DOR group exhibited significant enrichment in the choline metabolism and folate biosynthesis pathways, while the purine metabolism pathway was primarily enriched in the PCOS and NOR groups. Notably, the significantly different concentrations of pregnanediol-3-glucuronide and 2-hydroxyestrone sulfate in the DOR and NOR groups underscore their potential as biomarkers for assessing the occurrence of DOR [30]. Table 2 summarizes the identified metabolites with differential expression in the follicular fluid of women with diminished ovarian reserve.

### 3.3. Indicative Metabolomics Studies Investigating the Impact of Adjuvant Therapies in Follicular Fluid Metabolomic Profile of Women with POR

In 2023, He et al. conducted a prospective observational study to examine the impact of growth hormone (GH) as an adjuvant therapy on the follicular fluid metabolome of patients with diminished ovarian reserve (DOR) undergoing in vitro fertilization (IVF). This study compared the follicular fluid (FF) metabolomes of DOR patients who received GH co-treatment with those who did not, using gas chromatography-mass spectrometry (GC-MS) to identify metabolic profiles. A total of 134 metabolites were detected, with 24 showing significant concentration differences between the groups. In the GH-treated group, levels of itaconic acid, glutathione, cis-aconitic acid, N-alpha-acetyllysine, stearic acid, tridecane, and most organic acids were significantly increased, while S-adenosylmethionine (SAM), 2-oxobutyric acid, citramalic acid, and butylated hydroxytoluene levels were significantly decreased. Additionally, unsaturated fatty acids like linolelaidic acid, 9-heptadecenoic acid, and palmitelaidic acid were reduced in the GH group. Pearson correlation analysis further showed a positive correlation between the number of oocytes retrieved and levels of conjugated linoleic acid, itaconic acid, and tridecane, while D-norleucine and SAM levels were negatively correlated with oocyte retrieval. Enrichment analysis of differential metabolites, performed with the KEGG database, linked the identified metabolic changes to pathways involved in glutathione metabolism, cysteine and methionine metabolism, linoleic acid metabolism, unsaturated fatty acid biosynthesis, the tricarboxylic acid (TCA) cycle, glyoxylate metabolism, and lysine degradation. Based on these findings, the researchers suggested that GH co-treatment during controlled ovarian stimulation (COS) could enhance oocyte development by modifying the follicular fluid metabolite profiles in DOR patients. However, they noted that the observed downregulation of SAM, a critical regulator of genomic imprinting, may introduce a risk of imprinting disturbances, necessitating further investigation [31].

The 2023 study by Viardot-Foucault et al. aimed to examine metabolomic changes in the follicular fluid (FF) of patients with poor ovarian response (POR) following DHEA supplementation. FF samples were collected from POR patients undergoing IVF, comparing those supplemented with DHEA (DHEA+) to a control group without supplementation (DHEA−). Untargeted liquid chromatography–tandem mass spectrometry (LC-MS/MS) and a multiplex suspension immunoassay targeting 65 cytokines, chemokines, and growth factors were employed for metabolomic analysis. In total, 118 metabolites, including lipids, fatty acids, glucocorticoids, hormones, bile acids, peptides, and 22 cytokines, were identified in the FF of POR patients. Four metabolites—glycerophosphocholine, linoleic acid, progesterone, and L-valine—differed significantly between the DHEA+ and DHEA- groups, with levels notably lower in DHEA+ (*p* < 0.05–0.005). Correlation analysis in the DHEA+ group revealed that progesterone was positively associated with IGF-1 (Pearson r: 0.6757, *p* < 0.01), while glycerophosphocholine was negatively associated with AMH (Pearson r: −0.5815, *p* < 0.05). Linoleic acid correlated with both estradiol and IGF-1 (Pearson r: 0.7016 and 0.8203, *p* < 0.01), while valine in the DHEA- group negatively correlated with serum-free testosterone (Pearson r: −0.8774, *p* < 0.0001). Immunoassay analysis also revealed significantly lower MCP1, IFNγ, LIF, and VEGF-D levels in the DHEA+ group. In conclusion, this study provided novel insights into the FF metabolome in POR patients, showing that DHEA supplementation affects both the range and concentration of specific metabolites. The findings suggest possible mechanisms of DHEA metabolism and indicate that glycerophosphocholine, linoleic acid, progesterone, and L-valine may serve as potential biomarkers to assess DHEA supplementation effects [32]. Table 3 summarizes the identified metabolites with differential expression in the follicular fluid of women with diminished ovarian reserve in association with adjuvant therapies.

## 4. Discussion

Poor ovarian response represents one of the most significant challenges in assisted reproductive treatments due to its profound impact on the likelihood of successful in vitro fertilization (IVF) outcomes. Poor response is strongly associated with poor or diminished ovarian reserve, characterized by a reduced number of ovarian follicles and a limited capacity to produce high-quality oocytes. As a result, the number of embryos available for implantation is often insufficient, thereby reducing the probability of achieving a successful pregnancy. Current research is investigating various supplements and adjuvants, including dehydroepiandrosterone (DHEA), Coenzyme Q10, and growth hormone and innovative treatment strategies, such as stem cell therapy and platelet-rich plasma (PRP) intra-ovarian infusion, as potential interventions to enhance ovarian response or oocyte quality [8,33,34]. However, the evidence supporting their efficacy remains inconclusive [35,36]. Therefore, the need for further research to establish more effective and individualized strategies for predicting ovarian response and managing POR within the framework of assisted reproductive technology remains.

Metabolomics, combined with bioinformatics analysis, represents a high-throughput approach that enables comprehensive profiling of metabolite expression across various sample types, positioning it at the core of systems biology research. This powerful tool facilitates biomarker discovery and elucidates biochemical pathways and mechanisms underlying both physiological and pathological processes [37]. In recent years, the applications of omics, in general, has expanded significantly in the field of reproductive medicine, providing clinicians with precise, non-invasive, and personalized tools to enhance fertility outcomes in assisted reproductive technologies (ART) [20,38,39].

In this review, we discuss nine key metabolomic studies that analyze serum or follicular fluid samples in women with poor ovarian reserve (POR). Most of the included studies compared groups of women with normal ovarian reserve (NOR) to groups of patients with POR or diminished ovarian reserve (DOR), revealing significant differences in the expression of various metabolites following controlled ovarian stimulation (COS), in samples taken during egg retrieval, or on days 2–5 of the menstrual cycle without prior COS. Two studies specifically examined adjuvant therapies and the corresponding changes they cause in the metabolic profiles of patients with POR. The molecules identified across all of these studies span multiple categories, including phospholipids, fatty acids, triacylglycerols, methoxyphenols, steroids, amino acids, peptides, and other bioactive compounds.

Across both serum and follicular fluid samples, numerous metabolites and metabolic pathways potentially linked to poor ovarian response were identified. However, findings demonstrated significant variations between studies, likely due to differences in experimental designs and conditions. Additionally, the substantial variability in definitions of POR contributes to the lack of conclusive evidence and makes cross-study comparisons challenging. Despite these inconsistencies, some common findings have emerged. In particular, prostaglandin-related metabolites were detected in two of the studies, those of Liang et al. [29] and Song et al. [25], and were similarly downregulated in the POR/DOR groups compared to the NOR group.

Prostaglandins play a crucial role in regulating processes such as follicular maturation, cumulus cell expansion, ovulation, granulosa cell apoptosis, and protection against oxidative stress [40,41,42,43]. By supporting these mechanisms, they promote successful oocyte maturation and help preserve ovarian reserve and oocyte quality. Consequently, diminished prostaglandin levels may negatively affect both the quantity and quality of oocytes and serve as a potential indicator in women with POR. These metabolites, identified as significant for ovarian reserve and observed in two of the nine studies reviewed, could potentially serve as reliable biomarkers of ovarian function pending further research and validation.

A non-targeted metabonomic analysis of follicular fluid from DOR patients identified 12 upregulated and 32 downregulated metabolites, including amino acids, indoles, nucleosides, organic acids, steroids, phospholipids, fatty acyls, and organic oxygen compounds. A diagnostic model based on 10 key metabolites was proposed [44]. These metabolites were linked to aminoacyl-tRNA biosynthesis, tryptophan metabolism, pantothenate and CoA biosynthesis, and purine metabolism. Notably, pregnanediol-3-glucuronide, reported as downregulated in this study, was similarly identified as downregulated in the follicular fluid of DOR patients in the study by Shen et al. [30]. Additionally, L-aspartic acid, observed as upregulated in the current analysis, corresponds to findings by Song et al., who reported its upregulation in serum samples of women with POR [25].

Metabolic pathway analyses also revealed variability among studies. Serum analyses identified several pathways associated with POR, including nicotinate and nicotinamide metabolism, unsaturated fatty acid biosynthesis, linoleic acid metabolism, sphingolipid metabolism, aminoacyl-tRNA biosynthesis, alpha-linolenic acid metabolism, and the biosynthesis of phenylalanine, arginine, tyrosine, and tryptophan, as well as glycerophospholipid metabolism. Follicular fluid analyses highlighted pathways involved in cell proliferation, apoptosis, hormonal responses, and the arachidonic acid metabolic pathway. Similarly, in metabolomic analyses from granulosa cells of women with diminished ovarian response, steroid metabolites were found reduced in the granulosa cells [45]. Additionaly, in cumulus cells of young women with diminished ovarian response a de novo serine synthesis pathway was found increased [46].

The studies by He et al. and Viardot-Foucault et al. serve as illustrative examples of how metabolomics can be utilized to investigate the impact of adjuvant therapies on the follicular fluid (FF) metabolite composition in women with poor ovarian response (POR) [31,32]. These studies demonstrated that growth hormone (GH) and dehydroepiandrosterone (DHEA) induce alterations in specific FF metabolites, proposing potential mechanisms of action and identifying candidate biomarkers for evaluating the effects of supplementation. Such metabolomic studies hold promise for improving the management and monitoring of patients’ responses to adjuvant therapies for POR.

While many biomarkers have been proposed for poor or decreased ovarian response, further research is required to identify the optimal set of biomarkers, which must then be validated in multicenter, randomized trials to establish their clinical applicability in assisted reproduction. Moreover, establishing standardized guidelines for metabolomic and bioinformatic analyses globally is crucial for reducing discrepancies, improving consistency and reliability, and ultimately producing more reproducible findings in ovarian reserve and fertility research. While efforts to standardize clinical metabolomics are ongoing, continued collaboration among researchers and organizations worldwide is essential for refining these standards [47]. This collective effort holds great promise for enhancing the accuracy, reproducibility, and interpretability of metabolomic data, leading to more reliable outcomes in fertility studies and clinical applications. Furthermore, we propose that future studies integrating metabolomic analyses with other omics disciplines, particularly proteomics, have the potential to significantly expand the current body of knowledge and advance the investigation and management of poor ovarian response.

## 5. Conclusions

In conclusion, the results of metabolomic analyses conducted over the past decade have made substantial contributions to our understanding of the molecular mechanisms underlying poor ovarian response, diminished ovarian reserve, and oocyte development. However, the need for a standardized framework to guide the design of metabolomic and bioinformatics analysis experiments remains. Furthermore, additional studies are required to identify the optimal set of proposed biomarkers and to validate them through multicenter, prospective, and randomized controlled trials. Targeted omics research approaches could focus on metabolites associated with prostaglandins. Metabolomic data offer significant potential for identifying biomarkers that could enhance the prediction and management of ovarian response in patients with poor ovarian response (POR), potentially leading to improvements in oocyte quality and fertility outcomes.

## Figures and Tables

**Table 1:** Summary of metabolites with differential expression in the serum of women with poor or decreased ovarian reserve.

Metabolite	Compared Groups	Altered Expression in Women with POR/DOR	Study
C_34_H_70_O_2_ (*m*/*z* 533.51975) Attribution: Fatty alcohols	PR samples vs. NR vs. HR	Upregulated in PR	Borges Jr. et al. [24]
C_29_H_57_N_5_O_12_ (*m*/*z* 685.43505) Attribution: Amino acids, peptides, and analogs	PR samples vs. NR vs. HR	Upregulated in PR	
C_59_H_90_O_4_ (*m*/*z* 880.7391) Attribution: Quinone and hydroquinone lipids	PR samples vs. NR vs. HR	Upregulated in PR	
C_34_H_56_O_9_ (*m*/*z* 631.3793) Attribution: Steroidal glycosides	PR samples vs. NR vs. HR	Upregulated in PR	
C_48_H_72_O_2_ (*m*/*z* 698.5985) Attribution: Quinone and hydroquinone lipids	PR samples vs. NR vs. HR	Downregulated in PR	
C_36_H_75_N (*m*/*z* 522.5934) Attribution: Tertiary amines	PR samples vs. NR vs. HR	Downregulated in PR	
C_48_H_72_O_2_ (*m*/*z* 698.6051) Attribution: Quinone and hydroquinone lipids	PR samples vs. NR vs. HR	Downregulated in PR	
C_55_H_102_O_6_ (*m*/*z* 876.79465) Attribution: Triradylcglycerols	PR samples vs. NR vs. HR	Downregulated in PR	
C_56_H_94_O_6_ (*m*/*z* 880.7497) Attribution: Triradylcglycerols	PR samples vs. NR vs. HR	Downregulated in PR	
C_17_H_26_O_3_ (*m*/*z* 296.2295) Attribution: Methoxyphenols	PR samples vs. NR vs. HR	Downregulated in PR	
Tetracosanoic acid	POR vs. NOR	Downregulated in POR	Song et al. [25]
Pyracarbolid	POR vs. NOR	Upregulated in POR	
Diphenylamine	POR vs. NOR	Upregulated in POR	
Lanosterin	POR vs. NOR	Upregulated in POR	
Pelargonic acid	POR vs. NOR	Upregulated in POR	
Sebacic acid	POR vs. NOR	Upregulated in POR	
2-arachidonoylglycerol	POR vs. NOR	Upregulated in POR	
Lidocaine	POR vs. NOR	Upregulated in POR	
Cortexolone	POR vs. NOR	Downregulated in POR	
Prostaglandin H2	POR vs. NOR	Downregulated in POR	
1-naphthylamine	POR vs. NOR	Upregulated in POR	
5-hydroxymethyl-2-furancarboxaldehyde	POR vs. NOR	Downregulated in POR	
2,4-dinitrophenol	POR vs. NOR	Upregulated in POR	
D-erythrulose1-phosphate	POR vs. NOR	Upregulated in POR	
L-aspartic acid	POR vs. NOR	Upregulated in POR	
6-hydroxynicotinate	POR vs. NOR	Upregulated in POR	
Maleic acid	POR vs. NOR	Downregulated in POR	
Succinic acid semialdehyde	POR vs. NOR	Downregulated in POR	
Stearic acid	DOR vs. NOR	Upregulated in DOR	An et al. [26]
Palmitic acid	DOR vs. NOR	Upregulated in DOR	
PC(18:0/9:0(CHO))	DOR vs. NOR	Downregulated in DOR	
PC(16:0/9:0(CHO))	DOR vs. NOR	Downregulated in DOR	
LysoPC(9:0(CHO)/0:0)	DOR vs. NOR	Downregulated in DOR	

PR: poor responders, NR: normal responders, HR: hyper-responders, POR: poor ovarian reserve, DOR: decreased ovarian reserve, NOR: normal ovarian reserve.

**Table 2:** Summary of metabolites with differential expression in the follicular fluid of women with poor or decreased ovarian reserve.

Metabolite	Compared Groups	Altered Expression in Women with POR/DOR	Study
C_48_H_88_NO_8_P (*m*/*z* 838.6785) Phosphatidylcholine subclass (PC)	POR vs. NOR	Upregulated in NOR	Cataldi et al. [27]
C_50_H_84_NO_8_P (*m*/*z* 858.5918) Phosphatidylcholine subclass (PC)	POR vs. NOR	Upregulated in NOR	
C_40_H_72_NO_8_P (*m*/*z* 726.5031) Phosphatidylcholine subclass (PC)	POR vs. NOR	Upregulated in NOR	
C_42_H_80_NO_8_P (*m*/*z* 834.4541) Phosphatidylethanolamines subclass (PE)	POR vs. NOR	Upregulated in POR	
C_42_H_77_O_10_P (*m*/*z* 811.4649) Phosphatidylglycerols subclass(PG)	POR vs. NOR	Upregulated in POR	
C_39_H_74_NO_8_P (*m*/*z* 716.5332) Phosphatidylethanolamines subclass (PE)	POR vs. NOR	Upregulated in POR	
C_41_H_75_O_13_P (*m*/*z* 807.4682) Phosphatidylinositols subclass (PI)	POR vs. NOR	Upregulated in POR	
C_47_H_72_O_5_ (*m*/*z* 739.5157) Diacylglycerols subclass (DAG)	POR vs. NOR	Upregulated in POR	
(*m*/*z* 844.4166) Not defined lipid subclass by HMDB database.	POR vs. NOR	Upregulated in POR	
C_38_H_76_NO_8_P (*m*/*z* 706.5328) Phosphatidylethanolamines subclass (PE)	POR vs. NOR	Upregulated in POR	
Polyunsaturated ChoPls (PUFA ae)	DOR vs. NOR	Downregulated in DOR	De La Barca et al. [28]
Unsaturated-to-Saturated choline plasmalogens (UFA /SFA ae) ratios	DOR vs. NOR	Downregulated in DOR	
Total Dimethylarginine-to-Arginine (Total DMA/Arginine)	DOR vs. NOR	Downregulated in DOR	
±20-HDoHE	DOR vs. NOR	Downregulated in DOR	Liang et al. [29]
±5-iso PGF2α-VI	DOR vs. NOR	Downregulated in DOR	
12S-HHTrE	DOR vs. NOR	Downregulated in DOR	
15-deoxy-Δ12,14-PGJ2	DOR vs. NOR	Downregulated in DOR	
1a,1b-dihomo PGE2	DOR vs. NOR	Downregulated in DOR	
1a,1b-dihomo PGF2α	DOR vs. NOR	Downregulated in DOR	
20-COOH-AA	DOR vs. NOR	Downregulated in DOR	
20-HETE	DOR vs. NOR	Downregulated in DOR	
8S,15S-DiHETE	DOR vs. NOR	Downregulated in DOR	
PGA2	DOR vs. NOR	Downregulated in DOR	
PGD2	DOR vs. NOR	Downregulated in DOR	
PGE1	DOR vs. NOR	Downregulated in DOR	
PGF1α	DOR vs. NOR	Downregulated in DOR	
PGF2α	DOR vs. NOR	Downregulated in DOR	
PGJ2	DOR vs. NOR	Downregulated in DOR	
(S)-nerolidol 3-O	DOR vs. NOR	Downregulated in DOR	Shen et al. [30]
S-japonin	DOR vs. NOR	Downregulated in DOR	
2-hydroxyestrone sulfate	DOR vs. NOR	Downregulated in DOR	
Pregnanediol-3-glucuronide	DOR vs. NOR	Downregulated in DOR	
3-O-acetylepisamarcandin	DOR vs. NOR	Downregulated in DOR	
Isopropyl linoleate	DOR vs. NOR	Upregulated in DOR	
DG (18:0/18:2(9Z,12Z)/0:0)	DOR vs. NOR	Upregulated in DOR	
Mactraxanthin	DOR vs. NOR	Upregulated in DOR	
DG (18:0/16:1(9Z)/0:0)	DOR vs. NOR	Upregulated in DOR	
PE (16:1(9Z)/P-18:1(11Z))	DOR vs. NOR	Upregulated in DOR	

POR: poor ovarian reserve, DOR: decreased ovarian reserve, NOR: normal ovarian reserve.

**Table 3:** Summary of metabolites with differential expression in association with adjuvant therapies.

Metabolite	Type of Sample	Compared Groups	Altered Expression in Women with POR/DOR	Study
Itaconic acid	Follicular Fluid	DOR GH group vs. DOR control	Upregulated in GH group	He et al. [31]
Glutathione	Follicular Fluid	DOR GH group vs. DOR control	Upregulated in GH group	
cis-Aconitic acid	Follicular Fluid	DOR GH group vs. DOR control	Upregulated in GH group	
N-alpha-acetyllysine	Follicular Fluid	DOR GH group vs. DOR control	Upregulated in GH group	
Stearic acid	Follicular Fluid	DOR GH group vs. DOR control	Upregulated in GH group	
Tridecane	Follicular Fluid	DOR GH group vs. DOR control	Upregulated in GH group	
3,6-Dianhydro-d-glucopyranose	Follicular Fluid	DOR GH group vs. DOR control	Upregulated in GH group	
Cyclotetrasiloxane	Follicular Fluid	DOR GH group vs. DOR control	Upregulated in GH group	
3H-Pyrazol-3-one, 2,4-dihydro	Follicular Fluid	DOR GH group vs. DOR control	Upregulated in GH group	
Bis(N-methoxy-N-methylamino)methane	Follicular Fluid	DOR GH group vs. DOR control	Upregulated in GH group	
Benzoic acid	Follicular Fluid	DOR GH group vs. DOR control	Upregulated in GH group	
1-Aziridineethanol	Follicular Fluid	DOR GH group vs. DOR control	Upregulated in GH group	
N-alpha-Acetyllycine	Follicular Fluid	DOR GH group vs. DOR control	Upregulated in GH group	
Conjugated linoleic acid	Follicular Fluid	DOR GH group vs. DOR control	Upregulated in GH group	
S-adenosylme- thionine (SAM)	Follicular Fluid	DOR GH group vs. DOR control	Downregulated in GH group	
2-oxobutyric acid	Follicular Fluid	DOR GH group vs. DOR control	Downregulated in GH group	
Citramalic acid	Follicular Fluid	DOR GH group vs. DOR control	Downregulated in GH group	
Butylated hydroxytoluene	Follicular Fluid	DOR GH group vs. DOR control	Downregulated in GH group	
Linolelaidic acid	Follicular Fluid	DOR GH group vs. DOR control	Downregulated in GH group	
9-Heptadecenoic Acid	Follicular Fluid	DOR GH group vs. DOR control	Downregulated in GH group	
Palmitelaidic acid	Follicular Fluid	DOR GH group vs. DOR control	Downregulated in GH group	
Lysine	Follicular Fluid	DOR GH group vs. DOR control	Downregulated in GH group	
3-Pentenoic acid, 4-methyl	Follicular Fluid	DOR GH group vs. DOR control	Downregulated in GH group	
D-Norleucine, N-methoxycarbonyl	Follicular Fluid	DOR GH group vs. DOR control	Downregulated in GH group	
2,4-Imidazolidinenedione, 1-methyl	Follicular Fluid	DOR GH group vs. DOR control	Downregulated in GH group	
Glycerophosphocholine	Follicular Fluid	POR DHEA+ vs. POR DHEA-	Downregulated in DHEA+ group	Viardot-Foucault et al. [32]
Linoleic acid	Follicular Fluid	POR DHEA+ vs. POR DHEA-	Downregulated in DHEA+ group	
Progesterone	Follicular Fluid	POR DHEA+ vs. POR DHEA-	Downregulated in DHEA+ group	
L-valine	Follicular Fluid	POR DHEA+ vs. POR DHEA-	Downregulated in DHEA+ group	
Cortisol	Follicular Fluid	POR DHEA+ vs. POR DHEA-	Upregulated in DHEA+ group	
MCP1	Follicular Fluid	POR DHEA+ vs. POR DHEA-	Downregulated in DHEA+ group	
IFNγ	Follicular Fluid	POR DHEA+ vs. POR DHEA-	Downregulated in DHEA+ group	
LIF	Follicular Fluid	POR DHEA+ vs. POR DHEA-	Downregulated in DHEA+ group	
VEGF-D	Follicular Fluid	POR DHEA+ vs. POR DHEA-	Downregulated in DHEA+ group	

POR: poor ovarian reserve, DOR: decreased ovarian reserve, GH: growth hormone, DHEA: dehydroepiandrosterone.

## Data Availability

No new data were created or analyzed in this study.
